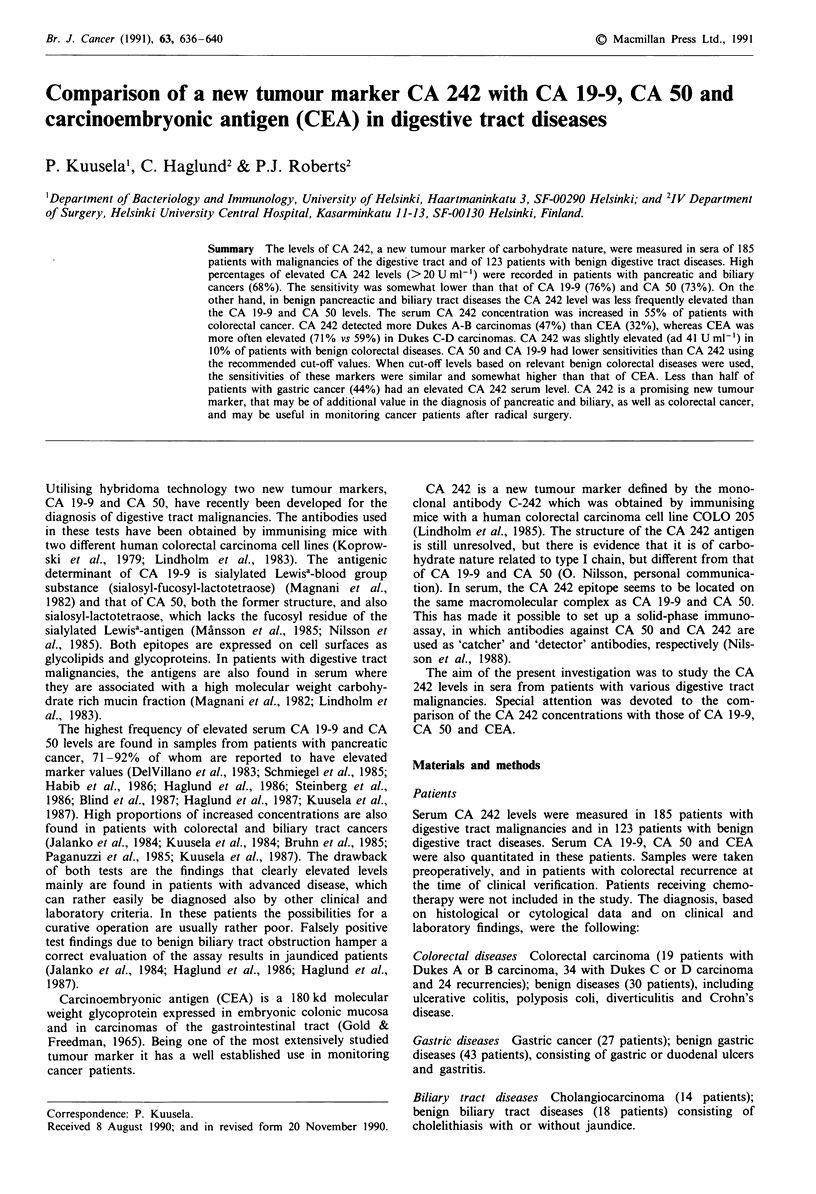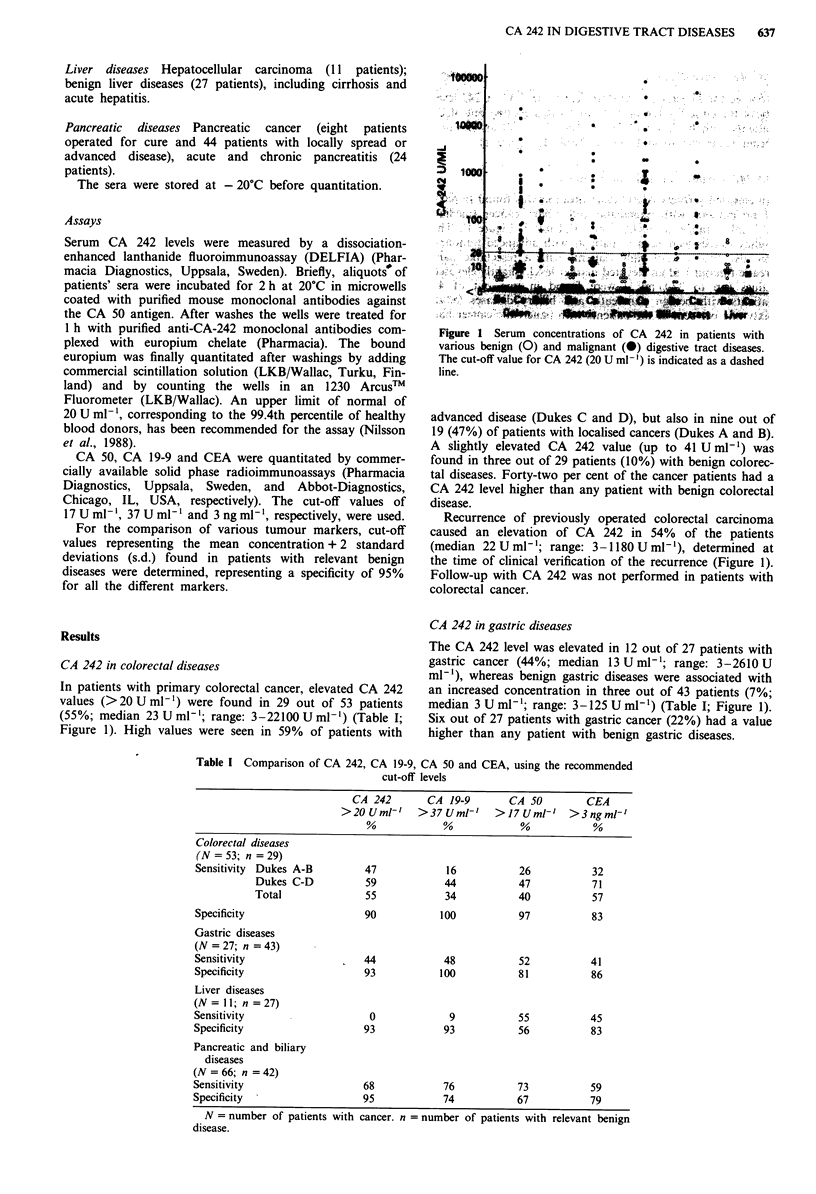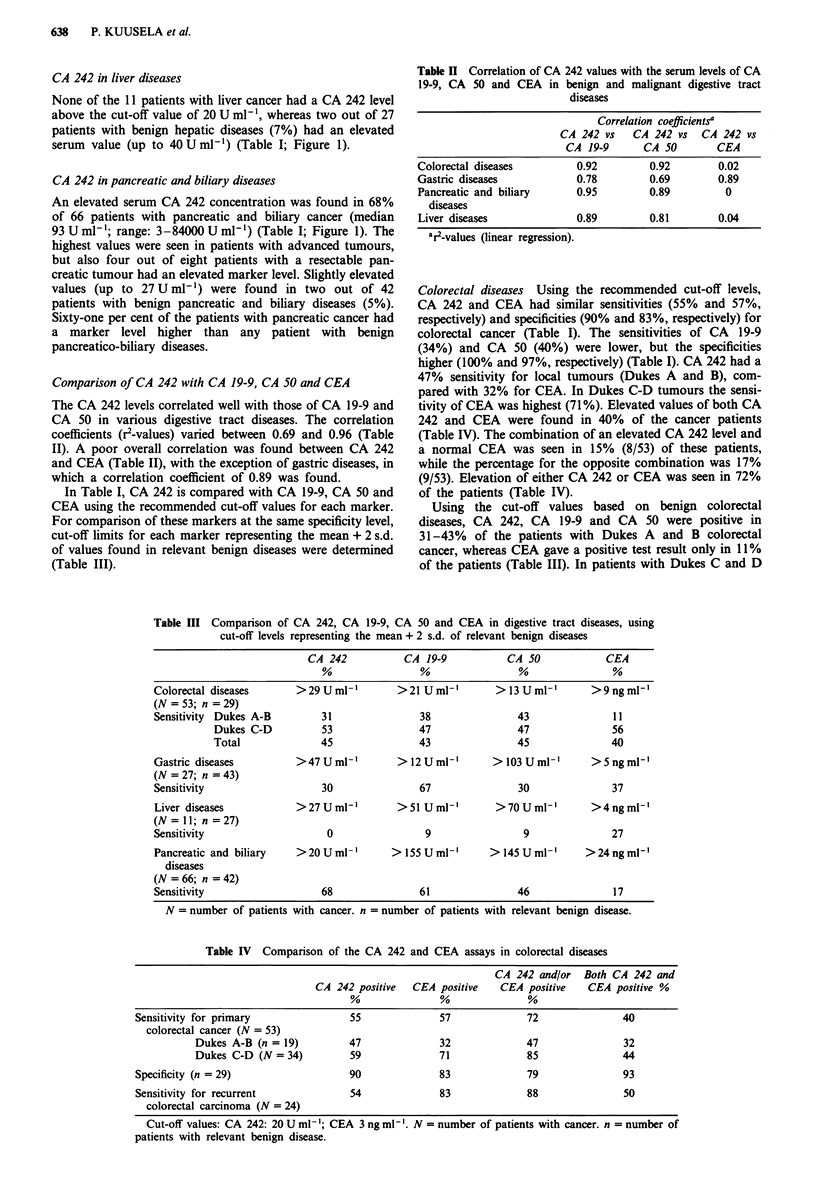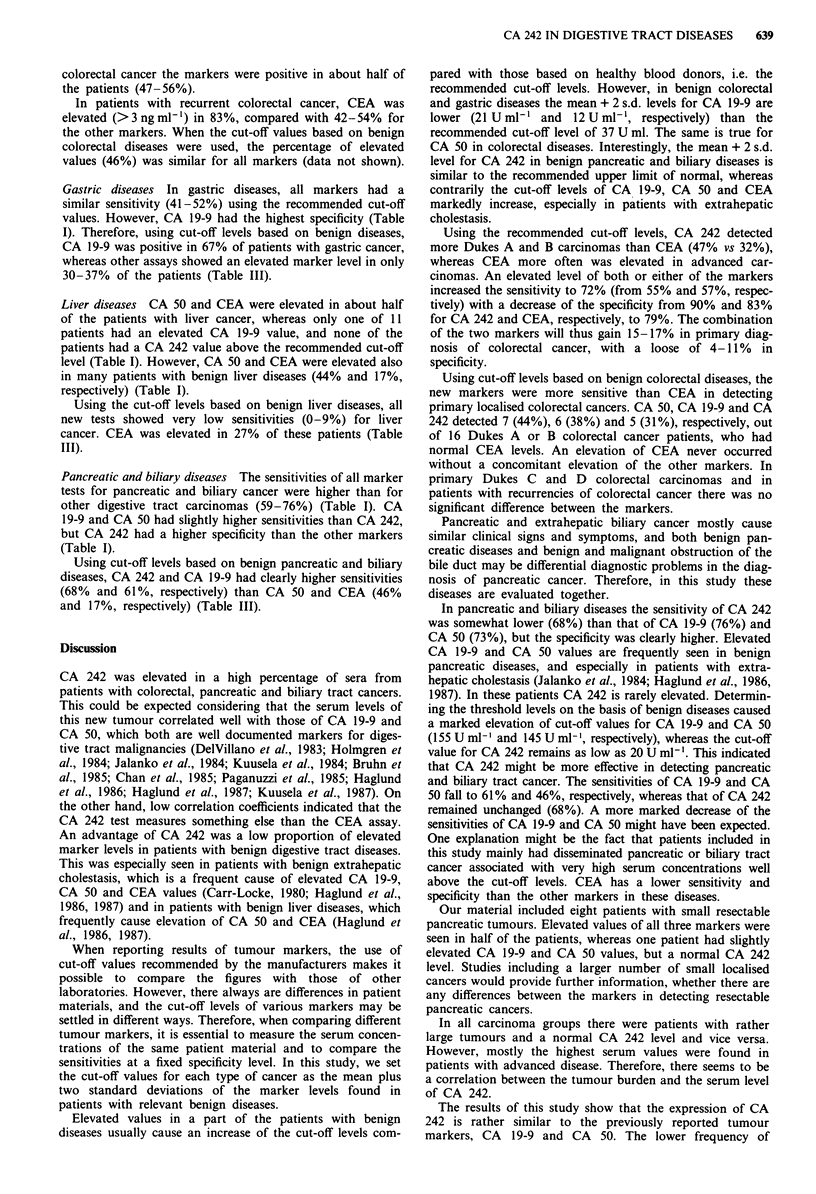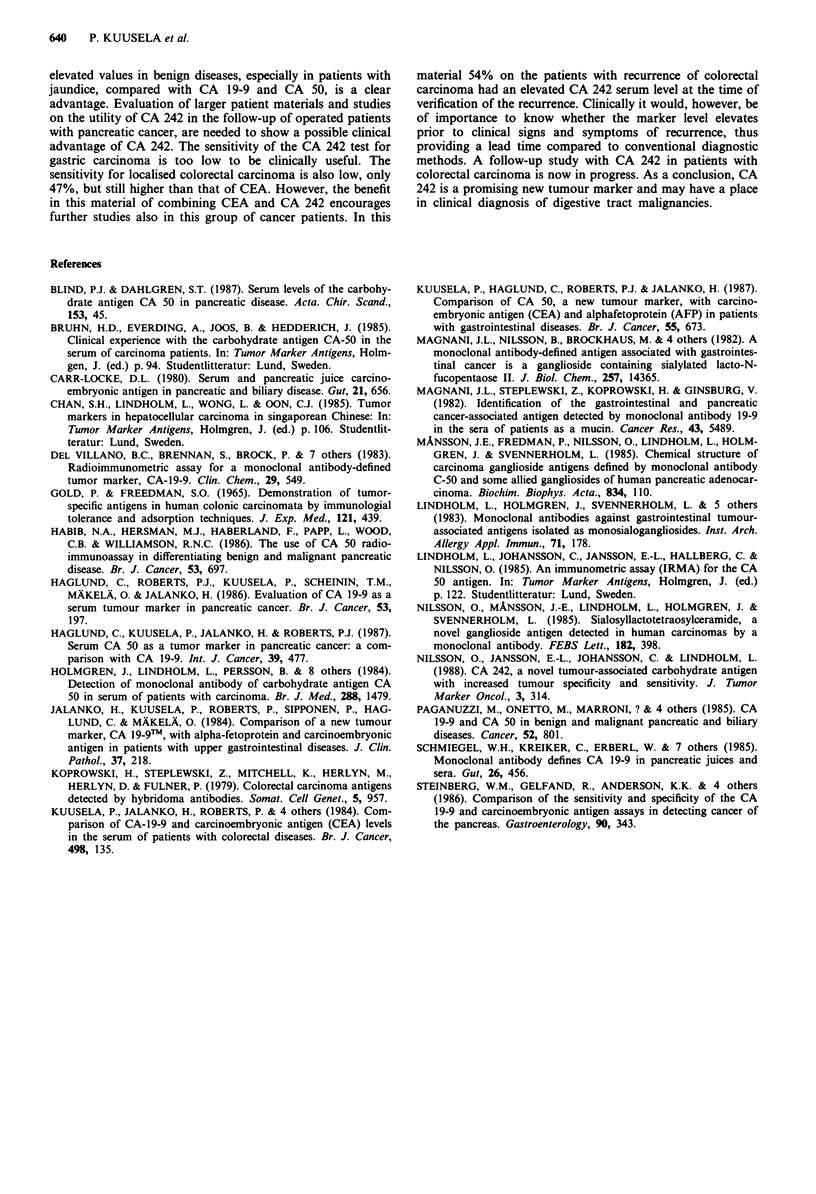# Comparison of a new tumour marker CA 242 with CA 19-9, CA 50 and carcinoembryonic antigen (CEA) in digestive tract diseases.

**DOI:** 10.1038/bjc.1991.146

**Published:** 1991-04

**Authors:** P. Kuusela, C. Haglund, P. J. Roberts

**Affiliations:** Department of Bacteriology and Immunology, University of Helsinki, Finland.

## Abstract

The levels of CA 242, a new tumour marker of carbohydrate nature, were measured in sera of 185 patients with malignancies of the digestive tract and of 123 patients with benign digestive tract diseases. High percentages of elevated CA 242 levels (greater than 20 U ml-1) were recorded in patients with pancreatic and biliary cancers (68%). The sensitivity was somewhat lower than that of CA 19-9 (76%) and CA 50 (73%). On the other hand, in benign pancreatic and biliary tract diseases the CA 242 level was less frequently elevated than the CA 19-9 and CA 50 levels. The serum CA 242 concentration was increased in 55% of patients with colorectal cancer. CA 242 detected more Dukes A-B carcinomas (47%) than CEA (32%), whereas CEA was more often elevated (71% vs 59%) in Dukes C-D carcinomas. CA 242 was slightly elevated (ad 41 U ml-1) in 10% of patients with benign colorectal diseases. CA 50 and CA 19-9 had lower sensitivities than CA 242 using the recommended cut-off values. When cut-off levels based on relevant benign colorectal diseases were used, the sensitivities of these markers were similar and somewhat higher than that of CEA. Less than half of patients with gastric cancer (44%) had an elevated CA 242 serum level. CA 242 is a promising new tumour marker, that may be of additional value in the diagnosis of pancreatic and biliary, as well as colorectal cancer, and may be useful in monitoring cancer patients after radical surgery.


Br. J. Cancer (1991), 63, 636-640 © Macmillan Press Ltd., 1991

### Comparison of a new tumour marker CA 242 with CA 19-9, CA 50 and carcinoembryonic antigen (CEA) in digestive tract diseases

P. Kuusela[1], C. Haglund[2] & P.J. Roberts[2]

[1]*Department of Bacteriology and Immunology, University of Helsinki, Haartmaninkatu 3, SF-00290 Helsinki; and* [2]*IV Department of Surgery, Helsinki University Central Hospital, Kasarminkatu 11-13, SF-00130 Helsinki, Finland.*

**Summary** The levels of CA 242, a new tumour marker of carbohydrate nature, were measured in sera of 185 patients with malignancies of the digestive tract and of 123 patients with benign digestive tract diseases. High percentages of elevated CA 242 levels (>20 U ml$^{-1}$) were recorded in patients with pancreatic and biliary cancers (68%). The sensitivity was somewhat lower than that of CA 19-9 (76%) and CA 50 (73%). On the other hand, in benign pancreactic and biliary tract diseases the CA 242 level was less frequently elevated than the CA 19-9 and CA 50 levels. The serum CA 242 concentration was increased in 55% of patients with colorectal cancer. CA 242 detected more Dukes A-B carcinomas (47%) than CEA (32%), whereas CEA was more often elevated (71% *vs* 59%) in Dukes C-D carcinomas. CA 242 was slightly elevated (ad 41 U ml$^{-1}$) in 10% of patients with benign colorectal diseases. CA 50 and CA 19-9 had lower sensitivities than CA 242 using the recommended cut-off values. When cut-off levels based on relevant benign colorectal diseases were used, the sensitivities of these markers were similar and somewhat higher than that of CEA. Less than half of patients with gastric cancer (44%) had an elevated CA 242 serum level. CA 242 is a promising new tumour marker, that may be of additional value in the diagnosis of pancreatic and biliary, as well as colorectal cancer, and may be useful in monitoring cancer patients after radical surgery.

Utilising hybridoma technology two new tumour markers, CA 19-9 and CA 50, have recently been developed for the diagnosis of digestive tract malignancies. The antibodies used in these tests have been obtained by immunising mice with two different human colorectal carcinoma cell lines (Koprowski *et al.*, 1979; Lindholm *et al.*, 1983). The antigenic determinant of CA 19-9 is sialylated Lewis$^a$-blood group substance (sialosyl-fucosyl-lactotetraose) (Magnani *et al.*, 1982) and that of CA 50, both the former structure, and also sialosyl-lactotetraose, which lacks the fucosyl residue of the sialylated Lewis$^a$-antigen (Månsson *et al.*, 1985; Nilsson *et al.*, 1985). Both epitopes are expressed on cell surfaces as glycolipids and glycoproteins. In patients with digestive tract malignancies, the antigens are also found in serum where they are associated with a high molecular weight carbohydrate rich mucin fraction (Magnani *et al.*, 1982; Lindholm *et al.*, 1983).

The highest frequency of elevated serum CA 19-9 and CA 50 levels are found in samples from patients with pancreatic cancer, 71–92% of whom are reported to have elevated marker values (DelVillano *et al.*, 1983; Schmiegel *et al.*, 1985; Habib *et al.*, 1986; Haglund *et al.*, 1986; Steinberg *et al.*, 1986; Blind *et al.*, 1987; Haglund *et al.*, 1987; Kuusela *et al.*, 1987). High proportions of increased concentrations are also found in patients with colorectal and biliary tract cancers (Jalanko *et al.*, 1984; Kuusela *et al.*, 1984; Bruhn *et al.*, 1985; Paganuzzi *et al.*, 1985; Kuusela *et al.*, 1987). The drawback of both tests are the findings that clearly elevated levels mainly are found in patients with advanced disease, which can rather easily be diagnosed also by other clinical and laboratory criteria. In these patients the possibilities for a curative operation are usually rather poor. Falsely positive test findings due to benign biliary tract obstruction hamper a correct evaluation of the assay results in jaundiced patients (Jalanko *et al.*, 1984; Haglund *et al.*, 1986; Haglund *et al.*, 1987).

Carcinoembryonic antigen (CEA) is a 180 kd molecular weight glycoprotein expressed in embryonic colonic mucosa and in carcinomas of the gastrointestinal tract (Gold & Freedman, 1965). Being one of the most extensively studied tumour marker it has a well established use in monitoring cancer patients.

Correspondence: P. Kuusela.
Received 8 August 1990; and in revised form 20 November 1990.

CA 242 is a new tumour marker defined by the monoclonal antibody C-242 which was obtained by immunising mice with a human colorectal carcinoma cell line COLO 205 (Lindholm *et al.*, 1985). The structure of the CA 242 antigen is still unresolved, but there is evidence that it is of carbohydrate nature related to type I chain, but different from that of CA 19-9 and CA 50 (O. Nilsson, personal communication). In serum, the CA 242 epitope seems to be located on the same macromolecular complex as CA 19-9 and CA 50. This has made it possible to set up a solid-phase immunoassay, in which antibodies against CA 50 and CA 242 are used as 'catcher' and 'detector' antibodies, respectively (Nilsson *et al.*, 1988).

The aim of the present investigation was to study the CA 242 levels in sera from patients with various digestive tract malignancies. Special attention was devoted to the comparison of the CA 242 concentrations with those of CA 19-9, CA 50 and CEA.

### Materials and methods

### *Patients*

Serum CA 242 levels were measured in 185 patients with digestive tract malignancies and in 123 patients with benign digestive tract diseases. Serum CA 19-9, CA 50 and CEA were also quantitated in these patients. Samples were taken preoperatively, and in patients with colorectal recurrence at the time of clinical verification. Patients receiving chemotherapy were not included in the study. The diagnosis, based on histological or cytological data and on clinical and laboratory findings, were the following:

*Colorectal diseases* Colorectal carcinoma (19 patients with Dukes A or B carcinoma, 34 with Dukes C or D carcinoma and 24 recurrencies); benign diseases (30 patients), including ulcerative colitis, polyposis coli, diverticulitis and Crohn's disease.

*Gastric diseases* Gastric cancer (27 patients); benign gastric diseases (43 patients), consisting of gastric or duodenal ulcers and gastritis.

*Biliary tract diseases* Cholangiocarcinoma (14 patients); benign biliary tract diseases (18 patients) consisting of cholelithiasis with or without jaundice.

*Liver diseases* Hepatocellular carcinoma (11 patients); benign liver diseases (27 patients), including cirrhosis and acute hepatitis.

*Pancreatic diseases* Pancreatic cancer (eight patients operated for cure and 44 patients with locally spread or advanced disease), acute and chronic pancreatitis (24 patients).

The sera were stored at − 20°C before quantitation.

### *Assays*

Serum CA 242 levels were measured by a dissociation-enhanced lanthanide fluoroimmunoassay (DELFIA) (Pharmacia Diagnostics, Uppsala, Sweden). Briefly, aliquots of patients' sera were incubated for 2 h at 20°C in microwells coated with purified mouse monoclonal antibodies against the CA 50 antigen. After washes the wells were treated for 1 h with purified anti-CA-242 monoclonal antibodies complexed with europium chelate (Pharmacia). The bound europium was finally quantitated after washings by adding commercial scintillation solution (LKB/Wallac, Turku, Finland) and by counting the wells in an 1230 Arcus™ Fluorometer (LKB/Wallac). An upper limit of normal of 20 U $ml^{-1}$, corresponding to the 99.4th percentile of healthy blood donors, has been recommended for the assay (Nilsson *et al.*, 1988).

CA 50, CA 19-9 and CEA were quantitated by commercially available solid phase radioimmunoassays (Pharmacia Diagnostics, Uppsala, Sweden, and Abbot-Diagnostics, Chicago, IL, USA, respectively). The cut-off values of 17 U $ml^{-1}$, 37 U $ml^{-1}$ and 3 ng $ml^{-1}$, respectively, were used.

For the comparison of various tumour markers, cut-off values representing the mean concentration + 2 standard deviations (s.d.) found in patients with relevant benign diseases were determined, representing a specificity of 95% for all the different markers.

### Results

### *CA 242 in colorectal diseases*

In patients with primary colorectal cancer, elevated CA 242 values (>20 U $ml^{-1}$) were found in 29 out of 53 patients (55%; median 23 U $ml^{-1}$; range: 3–22100 U $ml^{-1}$) (Table I; Figure 1). High values were seen in 59% of patients with advanced disease (Dukes C and D), but also in nine out of 19 (47%) of patients with localised cancers (Dukes A and B). A slightly elevated CA 242 value (up to 41 U $ml^{-1}$) was found in three out of 29 patients (10%) with benign colorectal diseases. Forty-two per cent of the cancer patients had a CA 242 level higher than any patient with benign colorectal disease.

**Figure 1** Serum concentrations of CA 242 in patients with various benign (○) and malignant (●) digestive tract diseases. The cut-off value for CA 242 (20 U $ml^{-1}$) is indicated as a dashed line.

Recurrence of previously operated colorectal carcinoma caused an elevation of CA 242 in 54% of the patients (median 22 U $ml^{-1}$; range: 3–1180 U $ml^{-1}$), determined at the time of clinical verification of the recurrence (Figure 1). Follow-up with CA 242 was not performed in patients with colorectal cancer.

### *CA 242 in gastric diseases*

The CA 242 level was elevated in 12 out of 27 patients with gastric cancer (44%; median 13 U $ml^{-1}$; range: 3–2610 U $ml^{-1}$), whereas benign gastric diseases were associated with an increased concentration in three out of 43 patients (7%; median 3 U $ml^{-1}$; range: 3–125 U $ml^{-1}$) (Table I; Figure 1). Six out of 27 patients with gastric cancer (22%) had a value higher than any patient with benign gastric diseases.

**Table I** Comparison of CA 242, CA 19-9, CA 50 and CEA, using the recommended cut-off levels

| | | *CA 242* >*20 U ml⁻¹* % | *CA 19-9* >*37 U ml⁻¹* % | *CA 50* >*17 U ml⁻¹* % | *CEA* >*3 ng ml⁻¹* % |
|---|---|---|---|---|---|
| *Colorectal diseases* (*N* = 53; *n* = 29) | | | | | |
| Sensitivity | Dukes A-B | 47 | 16 | 26 | 32 |
| | Dukes C-D | 59 | 44 | 47 | 71 |
| | Total | 55 | 34 | 40 | 57 |
| Specificity | | 90 | 100 | 97 | 83 |
| Gastric diseases (*N* = 27; *n* = 43) | | | | | |
| Sensitivity | | 44 | 48 | 52 | 41 |
| Specificity | | 93 | 100 | 81 | 86 |
| Liver diseases (*N* = 11; *n* = 27) | | | | | |
| Sensitivity | | 0 | 9 | 55 | 45 |
| Specificity | | 93 | 93 | 56 | 83 |
| Pancreatic and biliary diseases (*N* = 66; *n* = 42) | | | | | |
| Sensitivity | | 68 | 76 | 73 | 59 |
| Specificity | | 95 | 74 | 67 | 79 |

*N* = number of patients with cancer. *n* = number of patients with relevant benign disease.

*CA 242 in liver diseases*

None of the 11 patients with liver cancer had a CA 242 level above the cut-off value of 20 U $ml^{-1}$, whereas two out of 27 patients with benign hepatic diseases (7%) had an elevated serum value (up to 40 U $ml^{-1}$) (Table I; Figure 1).

*CA 242 in pancreatic and biliary diseases*

An elevated serum CA 242 concentration was found in 68% of 66 patients with pancreatic and biliary cancer (median 93 U $ml^{-1}$; range: 3–84000 U $ml^{-1}$) (Table I; Figure 1). The highest values were seen in patients with advanced tumours, but also four out of eight patients with a resectable pancreatic tumour had an elevated marker level. Slightly elevated values (up to 27 U $ml^{-1}$) were found in two out of 42 patients with benign pancreatic and biliary diseases (5%). Sixty-one per cent of the patients with pancreatic cancer had a marker level higher than any patient with benign pancreatico-biliary diseases.

*Comparison of CA 242 with CA 19-9, CA 50 and CEA*

The CA 242 levels correlated well with those of CA 19-9 and CA 50 in various digestive tract diseases. The correlation coefficients ($r^2$-values) varied between 0.69 and 0.96 (Table II). A poor overall correlation was found between CA 242 and CEA (Table II), with the exception of gastric diseases, in which a correlation coefficient of 0.89 was found.

In Table I, CA 242 is compared with CA 19-9, CA 50 and CEA using the recommended cut-off values for each marker. For comparison of these markers at the same specificity level, cut-off limits for each marker representing the mean + 2 s.d. of values found in relevant benign diseases were determined (Table III).

**Table II** Correlation of CA 242 values with the serum levels of CA 19-9, CA 50 and CEA in benign and malignant digestive tract diseases

| | *Correlation coefficients*[a] | | |
|---|---|---|---|
| | *CA 242 vs CA 19-9* | *CA 242 vs CA 50* | *CA 242 vs CEA* |
| Colorectal diseases | 0.92 | 0.92 | 0.02 |
| Gastric diseases | 0.78 | 0.69 | 0.89 |
| Pancreatic and biliary diseases | 0.95 | 0.89 | 0 |
| Liver diseases | 0.89 | 0.81 | 0.04 |

[a]$r^2$-values (linear regression).

*Colorectal diseases* Using the recommended cut-off levels, CA 242 and CEA had similar sensitivities (55% and 57%, respectively) and specificities (90% and 83%, respectively) for colorectal cancer (Table I). The sensitivities of CA 19-9 (34%) and CA 50 (40%) were lower, but the specificities higher (100% and 97%, respectively) (Table I). CA 242 had a 47% sensitivity for local tumours (Dukes A and B), compared with 32% for CEA. In Dukes C-D tumours the sensitivity of CEA was highest (71%). Elevated values of both CA 242 and CEA were found in 40% of the cancer patients (Table IV). The combination of an elevated CA 242 level and a normal CEA was seen in 15% (8/53) of these patients, while the percentage for the opposite combination was 17% (9/53). Elevation of either CA 242 or CEA was seen in 72% of the patients (Table IV).

Using the cut-off values based on benign colorectal diseases, CA 242, CA 19-9 and CA 50 were positive in 31–43% of the patients with Dukes A and B colorectal cancer, whereas CEA gave a positive test result only in 11% of the patients (Table III). In patients with Dukes C and D

**Table III** Comparison of CA 242, CA 19-9, CA 50 and CEA in digestive tract diseases, using cut-off levels representing the mean + 2 s.d. of relevant benign diseases

| | *CA 242* % | *CA 19-9* % | *CA 50* % | *CEA* % |
|---|---|---|---|---|
| Colorectal diseases (*N* = 53; *n* = 29) | >29 U $ml^{-1}$ | >21 U $ml^{-1}$ | >13 U $ml^{-1}$ | >9 ng $ml^{-1}$ |
| Sensitivity Dukes A-B | 31 | 38 | 43 | 11 |
| Dukes C-D | 53 | 47 | 47 | 56 |
| Total | 45 | 43 | 45 | 40 |
| Gastric diseases (*N* = 27; *n* = 43) | >47 U $ml^{-1}$ | >12 U $ml^{-1}$ | >103 U $ml^{-1}$ | >5 ng $ml^{-1}$ |
| Sensitivity | 30 | 67 | 30 | 37 |
| Liver diseases (*N* = 11; *n* = 27) | >27 U $ml^{-1}$ | >51 U $ml^{-1}$ | >70 U $ml^{-1}$ | >4 ng $ml^{-1}$ |
| Sensitivity | 0 | 9 | 9 | 27 |
| Pancreatic and biliary diseases (*N* = 66; *n* = 42) | >20 U $ml^{-1}$ | >155 U $ml^{-1}$ | >145 U $ml^{-1}$ | >24 ng $ml^{-1}$ |
| Sensitivity | 68 | 61 | 46 | 17 |

*N* = number of patients with cancer. *n* = number of patients with relevant benign disease.

**Table IV** Comparison of the CA 242 and CEA assays in colorectal diseases

| | *CA 242 positive* % | *CEA positive* % | *CA 242 and/or CEA positive* % | *Both CA 242 and CEA positive* % |
|---|---|---|---|---|
| Sensitivity for primary colorectal cancer (*N* = 53) | 55 | 57 | 72 | 40 |
| Dukes A-B (*n* = 19) | 47 | 32 | 47 | 32 |
| Dukes C-D (*N* = 34) | 59 | 71 | 85 | 44 |
| Specificity (*n* = 29) | 90 | 83 | 79 | 93 |
| Sensitivity for recurrent colorectal carcinoma (*N* = 24) | 54 | 83 | 88 | 50 |

Cut-off values: CA 242: 20 U $ml^{-1}$; CEA 3 ng $ml^{-1}$. *N* = number of patients with cancer. *n* = number of patients with relevant benign disease.

colorectal cancer the markers were positive in about half of the patients (47–56%).

In patients with recurrent colorectal cancer, CEA was elevated (>3 ng $ml^{-1}$) in 83%, compared with 42–54% for the other markers. When the cut-off values based on benign colorectal diseases were used, the percentage of elevated values (46%) was similar for all markers (data not shown).

*Gastric diseases* In gastric diseases, all markers had a similar sensitivity (41–52%) using the recommended cut-off values. However, CA 19-9 had the highest specificity (Table I). Therefore, using cut-off levels based on benign diseases, CA 19-9 was positive in 67% of patients with gastric cancer, whereas other assays showed an elevated marker level in only 30–37% of the patients (Table III).

*Liver diseases* CA 50 and CEA were elevated in about half of the patients with liver cancer, whereas only one of 11 patients had an elevated CA 19-9 value, and none of the patients had a CA 242 value above the recommended cut-off level (Table I). However, CA 50 and CEA were elevated also in many patients with benign liver diseases (44% and 17%, respectively) (Table I).

Using the cut-off levels based on benign liver diseases, all new tests showed very low sensitivities (0–9%) for liver cancer. CEA was elevated in 27% of these patients (Table III).

*Pancreatic and biliary diseases* The sensitivities of all marker tests for pancreatic and biliary cancer were higher than for other digestive tract carcinomas (59–76%) (Table I). CA 19-9 and CA 50 had slightly higher sensitivities than CA 242, but CA 242 had a higher specificity than the other markers (Table I).

Using cut-off levels based on benign pancreatic and biliary diseases, CA 242 and CA 19-9 had clearly higher sensitivities (68% and 61%, respectively) than CA 50 and CEA (46% and 17%, respectively) (Table III).

### Discussion

CA 242 was elevated in a high percentage of sera from patients with colorectal, pancreatic and biliary tract cancers. This could be expected considering that the serum levels of this new tumour correlated well with those of CA 19-9 and CA 50, which both are well documented markers for digestive tract malignancies (DelVillano *et al.*, 1983; Holmgren *et al.*, 1984; Jalanko *et al.*, 1984; Kuusela *et al.*, 1984; Bruhn *et al.*, 1985; Chan *et al.*, 1985; Paganuzzi *et al.*, 1985; Haglund *et al.*, 1986; Haglund *et al.*, 1987; Kuusela *et al.*, 1987). On the other hand, low correlation coefficients indicated that the CA 242 test measures something else than the CEA assay. An advantage of CA 242 was a low proportion of elevated marker levels in patients with benign digestive tract diseases. This was especially seen in patients with benign extrahepatic cholestasis, which is a frequent cause of elevated CA 19-9, CA 50 and CEA values (Carr-Locke, 1980; Haglund *et al.*, 1986, 1987) and in patients with benign liver diseases, which frequently cause elevation of CA 50 and CEA (Haglund *et al.*, 1986, 1987).

When reporting results of tumour markers, the use of cut-off values recommended by the manufacturers makes it possible to compare the figures with those of other laboratories. However, there always are differences in patient materials, and the cut-off levels of various markers may be settled in different ways. Therefore, when comparing different tumour markers, it is essential to measure the serum concentrations of the same patient material and to compare the sensitivities at a fixed specificity level. In this study, we set the cut-off values for each type of cancer as the mean plus two standard deviations of the marker levels found in patients with relevant benign diseases.

Elevated values in a part of the patients with benign diseases usually cause an increase of the cut-off levels compared with those based on healthy blood donors, i.e. the recommended cut-off levels. However, in benign colorectal and gastric diseases the mean + 2 s.d. levels for CA 19-9 are lower (21 U $ml^{-1}$ and 12 U $ml^{-1}$, respectively) than the recommended cut-off level of 37 U ml. The same is true for CA 50 in colorectal diseases. Interestingly, the mean + 2 s.d. level for CA 242 in benign pancreatic and biliary diseases is similar to the recommended upper limit of normal, whereas contrarily the cut-off levels of CA 19-9, CA 50 and CEA markedly increase, especially in patients with extrahepatic cholestasis.

Using the recommended cut-off levels, CA 242 detected more Dukes A and B carcinomas than CEA (47% *vs* 32%), whereas CEA more often was elevated in advanced carcinomas. An elevated level of both or either of the markers increased the sensitivity to 72% (from 55% and 57%, respectively) with a decrease of the specificity from 90% and 83% for CA 242 and CEA, respectively, to 79%. The combination of the two markers will thus gain 15–17% in primary diagnosis of colorectal cancer, with a loose of 4–11% in specificity.

Using cut-off levels based on benign colorectal diseases, the new markers were more sensitive than CEA in detecting primary localised colorectal cancers. CA 50, CA 19-9 and CA 242 detected 7 (44%), 6 (38%) and 5 (31%), respectively, out of 16 Dukes A or B colorectal cancer patients, who had normal CEA levels. An elevation of CEA never occurred without a concomitant elevation of the other markers. In primary Dukes C and D colorectal carcinomas and in patients with recurrencies of colorectal cancer there was no significant difference between the markers.

Pancreatic and extrahepatic biliary cancer mostly cause similar clinical signs and symptoms, and both benign pancreatic diseases and benign and malignant obstruction of the bile duct may be differential diagnostic problems in the diagnosis of pancreatic cancer. Therefore, in this study these diseases are evaluated together.

In pancreatic and biliary diseases the sensitivity of CA 242 was somewhat lower (68%) than that of CA 19-9 (76%) and CA 50 (73%), but the specificity was clearly higher. Elevated CA 19-9 and CA 50 values are frequently seen in benign pancreatic diseases, and especially in patients with extrahepatic cholestasis (Jalanko *et al.*, 1984; Haglund *et al.*, 1986, 1987). In these patients CA 242 is rarely elevated. Determining the threshold levels on the basis of benign diseases caused a marked elevation of cut-off values for CA 19-9 and CA 50 (155 U $ml^{-1}$ and 145 U $ml^{-1}$, respectively), whereas the cut-off value for CA 242 remains as low as 20 U $ml^{-1}$. This indicated that CA 242 might be more effective in detecting pancreatic and biliary tract cancer. The sensitivities of CA 19-9 and CA 50 fall to 61% and 46%, respectively, whereas that of CA 242 remained unchanged (68%). A more marked decrease of the sensitivities of CA 19-9 and CA 50 might have been expected. One explanation might be the fact that patients included in this study mainly had disseminated pancreatic or biliary tract cancer associated with very high serum concentrations well above the cut-off levels. CEA has a lower sensitivity and specificity than the other markers in these diseases.

Our material included eight patients with small resectable pancreatic tumours. Elevated values of all three markers were seen in half of the patients, whereas one patient had slightly elevated CA 19-9 and CA 50 values, but a normal CA 242 level. Studies including a larger number of small localised cancers would provide further information, whether there are any differences between the markers in detecting resectable pancreatic cancers.

In all carcinoma groups there were patients with rather large tumours and a normal CA 242 level and vice versa. However, mostly the highest serum values were found in patients with advanced disease. Therefore, there seems to be a correlation between the tumour burden and the serum level of CA 242.

The results of this study show that the expression of CA 242 is rather similar to the previously reported tumour markers, CA 19-9 and CA 50. The lower frequency of elevated values in benign diseases, especially in patients with jaundice, compared with CA 19-9 and CA 50, is a clear advantage. Evaluation of larger patient materials and studies on the utility of CA 242 in the follow-up of operated patients with pancreatic cancer, are needed to show a possible clinical advantage of CA 242. The sensitivity of the CA 242 test for gastric carcinoma is too low to be clinically useful. The sensitivity for localised colorectal carcinoma is also low, only 47%, but still higher than that of CEA. However, the benefit in this material of combining CEA and CA 242 encourages further studies also in this group of cancer patients. In this material 54% on the patients with recurrence of colorectal carcinoma had an elevated CA 242 serum level at the time of verification of the recurrence. Clinically it would, however, be of importance to know whether the marker level elevates prior to clinical signs and symptoms of recurrence, thus providing a lead time compared to conventional diagnostic methods. A follow-up study with CA 242 in patients with colorectal carcinoma is now in progress. As a conclusion, CA 242 is a promising new tumour marker and may have a place in clinical diagnosis of digestive tract malignancies.

### References

BLIND, P.J. & DAHLGREN, S.T. (1987). Serum levels of the carbohydrate antigen CA 50 in pancreatic disease. *Acta. Chir. Scand.*, **153**, 45.

BRUHN, H.D., EVERDING, A., JOOS, B. & HEDDERICH, J. (1985). Clinical experience with the carbohydrate antigen CA-50 in the serum of carcinoma patients. In: *Tumor Marker Antigens*, Holmgen, J. (ed.) p. 94. Studentlitteratur: Lund, Sweden.

CARR-LOCKE, D.L. (1980). Serum and pancreatic juice carcinoembryonic antigen in pancreatic and biliary disease. *Gut*, **21**, 656.

CHAN, S.H., LINDHOLM, L., WONG, L. & OON, C.J. (1985). Tumor markers in hepatocellular carcinoma in singaporean Chinese: In: *Tumor Marker Antigens*, Holmgren, J. (ed.) p. 106. Studentlitteratur: Lund, Sweden.

DEL VILLANO, B.C., BRENNAN, S., BROCK, P. & 7 others (1983). Radioimmunometric assay for a monoclonal antibody-defined tumor marker, CA-19-9. *Clin. Chem.*, **29**, 549.

GOLD, P. & FREEDMAN, S.O. (1965). Demonstration of tumor-specific antigens in human colonic carcinomata by immunologial tolerance and adsorption techniques. *J. Exp. Med.*, **121**, 439.

HABIB, N.A., HERSMAN, M.J., HABERLAND, F., PAPP, L., WOOD, C.B. & WILLIAMSON, R.N.C. (1986). The use of CA 50 radioimmunoassay in differentiating benign and malignant pancreatic disease. *Br. J. Cancer*, **53**, 697.

HAGLUND, C., ROBERTS, P.J., KUUSELA, P., SCHEININ, T.M., MÄKELÄ, O. & JALANKO, H. (1986). Evaluation of CA 19-9 as a serum tumour marker in pancreatic cancer. *Br. J. Cancer*, **53**, 197.

HAGLUND, C., KUUSELA, P., JALANKO, H. & ROBERTS, P.J. (1987). Serum CA 50 as a tumor marker in pancreatic cancer: a comparison with CA 19-9. *Int. J. Cancer*, **39**, 477.

HOLMGREN, J., LINDHOLM, L., PERSSON, B. & 8 others (1984). Detection of monoclonal antibody of carbohydrate antigen CA 50 in serum of patients with carcinoma. *Br. J. Med.*, **288**, 1479.

JALANKO, H., KUUSELA, P., ROBERTS, P., SIPPONEN, P., HAGLUND, C. & MÄKELÄ, O. (1984). Comparison of a new tumour marker, CA 19-9™, with alpha-fetoprotein and carcinoembryonic antigen in patients with upper gastrointestinal diseases. *J. Clin. Pathol.*, **37**, 218.

KOPROWSKI, H., STEPLEWSKI, Z., MITCHELL, K., HERLYN, M., HERLYN, D. & FULNER, P. (1979). Colorectal carcinoma antigens detected by hybridoma antibodies. *Somat. Cell Genet.*, **5**, 957.

KUUSELA, P., JALANKO, H., ROBERTS, P. & 4 others (1984). Comparison of CA-19-9 and carcinoembryonic antigen (CEA) levels in the serum of patients with colorectal diseases. *Br. J. Cancer*, **498**, 135.

KUUSELA, P., HAGLUND, C., ROBERTS, P.J. & JALANKO, H. (1987). Comparison of CA 50, a new tumour marker, with carcinoembryonic antigen (CEA) and alphafetoprotein (AFP) in patients with gastrointestinal diseases. *Br. J. Cancer*, **55**, 673.

MAGNANI, J.L., NILSSON, B., BROCKHAUS, M. & 4 others (1982). A monoclonal antibody-defined antigen associated with gastrointestinal cancer is a ganglioside containing sialylated lacto-N-fucopentaose II. *J. Biol. Chem.*, **257**, 14365.

MAGNANI, J.L., STEPLEWSKI, Z., KOPROWSKI, H. & GINSBURG, V. (1982). Identification of the gastrointestinal and pancreatic cancer-associated antigen detected by monoclonal antibody 19-9 in the sera of patients as a mucin. *Cancer Res.*, **43**, 5489.

MÅNSSON, J.E., FREDMAN, P., NILSSON, O., LINDHOLM, L., HOLMGREN, J. & SVENNERHOLM, L. (1985). Chemical structure of carcinoma ganglioside antigens defined by monoclonal antibody C-50 and some allied gangliosides of human pancreatic adenocarcinoma. *Biochim. Biophys. Acta.*, **834**, 110.

LINDHOLM, L., HOLMGREN, J., SVENNERHOLM, L. & 5 others (1983). Monoclonal antibodies against gastrointestinal tumour-associated antigens isolated as monosialogangliosides. *Inst. Arch. Allergy Appl. Immun.*, **71**, 178.

LINDHOLM, L., JOHANSSON, C., JANSSON, E.-L., HALLBERG, C. & NILSSON, O. (1985). An immunometric assay (IRMA) for the CA 50 antigen. In: *Tumor Marker Antigens*, Holmgren, J. (ed.) p. 122. Studentlitteratur: Lund, Sweden.

NILSSON, O., MÅNSSON, J.-E., LINDHOLM, L., HOLMGREN, J. & SVENNERHOLM, L. (1985). Sialosyllactotetraosylceramide, a novel ganglioside antigen detected in human carcinomas by a monoclonal antibody. *FEBS Lett.*, **182**, 398.

NILSSON, O., JANSSON, E.-L., JOHANSSON, C. & LINDHOLM, L. (1988). CA 242, a novel tumour-associated carbohydrate antigen with increased tumour specificity and sensitivity. *J. Tumor Marker Oncol.*, **3**, 314.

PAGANUZZI, M., ONETTO, M., MARRONI, ? & 4 others (1985). CA 19-9 and CA 50 in benign and malignant pancreatic and biliary diseases. *Cancer*, **52**, 801.

SCHMIEGEL, W.H., KREIKER, C., ERBERL, W. & 7 others (1985). Monoclonal antibody defines CA 19-9 in pancreatic juices and sera. *Gut*, **26**, 456.

STEINBERG, W.M., GELFAND, R., ANDERSON, K.K. & 4 others (1986). Comparison of the sensitivity and specificity of the CA 19-9 and carcinoembryonic antigen assays in detecting cancer of the pancreas. *Gastroenterology*, **90**, 343.